# Impact of the spatial resolution of satellite remote sensing sensors in the quantification of total suspended sediment concentration: A case study in turbid waters of Northern Western Australia

**DOI:** 10.1371/journal.pone.0175042

**Published:** 2017-04-05

**Authors:** Passang Dorji, Peter Fearns

**Affiliations:** Remote Sensing and Satellite Research Group, Curtin University, Perth, Western Australia; Bristol University/Remote Sensing Solutions Inc., UNITED STATES

## Abstract

The impact of anthropogenic activities on coastal waters is a cause of concern because such activities add to the total suspended sediment (TSS) budget of the coastal waters, which have negative impacts on the coastal ecosystem. Satellite remote sensing provides a powerful tool in monitoring TSS concentration at high spatiotemporal resolution, but coastal managers should be mindful that the satellite-derived TSS concentrations are dependent on the satellite sensor’s radiometric properties, atmospheric correction approaches, the spatial resolution and the limitations of specific TSS algorithms. In this study, we investigated the impact of different spatial resolutions of satellite sensor on the quantification of TSS concentration in coastal waters of northern Western Australia. We quantified the TSS product derived from MODerate resolution Imaging Spectroradiometer (MODIS)-Aqua, Landsat-8 Operational Land Image (OLI), and WorldView-2 (WV2) at native spatial resolutions of 250 m, 30 m and 2 m respectively and coarser spatial resolution (resampled up to 5 km) to quantify the impact of spatial resolution on the derived TSS product in different turbidity conditions. The results from the study show that in the waters of high turbidity and high spatial variability, the high spatial resolution WV2 sensor reported TSS concentration as high as 160 mg L^-1^ while the low spatial resolution MODIS-Aqua reported a maximum TSS concentration of 23.6 mg L^-1^. Degrading the spatial resolution of each satellite sensor for highly spatially variable turbid waters led to variability in the TSS concentrations of 114.46%, 304.68% and 38.2% for WV2, Landsat-8 OLI and MODIS-Aqua respectively. The implications of this work are particularly relevant in the situation of compliance monitoring where operations may be required to restrict TSS concentrations to a pre-defined limit.

## Introduction

Global coastal marine ecology is at ever increasing risk because of the increase of impacts due to the demands of maritime trade, supporting population growth which necessitates land reclamation, maintenance and capital dredging for ports, dredging for offshore resources, and placing of sub-sea transport pipelines [1, 2]. The Australian economy is heavily dependent on maritime links because of its geographical remoteness from other continents. One third of its GDP is based on sea-borne trade, and the existing ports that support this high volume of shipping traffic require constant maintenance dredging of existing shipping channels and frequent large capital dredging projects [3]. The environmental effects of dredging on the costal marine ecology are diverse, with dredging potentially resulting in either partial reduction or complete loss of marine habitat through the physical removal of substratum biota from the sub-sea surface and immediate burial due to sedimentation of the dredged materials [4]. Further, increase in turbidity caused by dredging significantly attenuates the amount of light reaching the benthic habitat for primary productivity [5–7]. The environmental cost of dredging and the need for coastal development poses a challenge to environmental monitoring agencies, marine ecologists and coastal infrastructure developers who aim to find a balance between the two [4].

Coastal water quality monitoring of the effects of anthropogenic processes aims to provide immediate and appropriate responses, but often requires continuous ground based monitoring, which is typically resource intensive, to maintain and only provides information on limited specific geographical locations [8, 9]. The availability of satellite remote sensing platforms has provided coastal managers with tools and capabilities to effectively monitor the coastal environment at spatial and temporal scales previously unconceivable from the perspective of traditional *in situ* based observation methods [10]. Coastal water quality in the form of water turbidity or Total Suspended Sediment (TSS) concentration has been widely studied across diverse geographical locations [11–20] by using a suite of remote sensing sensors such as, Landsat [21–30], MEdium Resolution Imaging Spectrometer (MERIS) [7, 31–33], MODerate resolution Imaging Spectroradiometer (MODIS) [16, 17, 20, 29, 34–44], and Sea-viewing Wide Field-of-view Sensor (SeaWiFS) [13, 45–49]. In addition to these most commonly used and “free to ground” sensors, commercial high spatial resolution sensors such as Systèm Pour l’Observation de la Terra (SPOT) [22, 50, 51], IKONOS [14] and WorldView-2 (WV2) [52] are also employed to map the TSS.

The high spatial resolution commercial satellite sensors such as IKONOS, WV2, and GeoEye-1 can provide data at spatial resolutions of approximately 0.5 m—4.0 m with temporal resolutions of ~1–8 days [53]. The freely available remote sensing data of MODIS and MERIS from the National Aeronautics and Space Administration can provide near-daily TSS estimates at 250 m—300 m resolution and Landsat at 30 m but with a monitoring frequency of 16 days. Previous studies [1, 9, 54, 55] conducted in mapping TSS for water quality monitoring have studied the spatial extent of suspended sediment plumes using one or more satellite sensors and the common consensus is that the higher spatial resolution satellite sensors are able to resolve finer details of suspended sediment plumes while the lower spatial resolution sensors lose the finer details. However, only a few studies [54, 55] have been conducted to study the impact of using different spatial resolution sensors in estimation of TSS in sediment plumes where the water can be spatially variable in TSS concentration, even at sub-pixel level. Ody et al. [54] showed that in the Gulf of Lion, France, the variability in the TSS concentration at the turbid fronts and edges of the river plume was estimated to be around 7 mg L^-1^ and 10 mg L^-1^ for 250 m and 1.0 km spatial resolution respectively. Further, the lower spatial resolution sensor SERVI (Spinning Enhanced Visible and Infrared Imager) at 3.0 x 5 km^2^ was shown to have TSS concentration variability due to different spatial resolution were as high as 20 mg L^-1^. The two studies [54, 55] indicated that the quantification of TSS concentrations using remote sensing sensors are not only determined by the spatial resolution of the sensors, but also the TSS variability of the region itself. Generally, the coarser spatial resolution sensors would produce higher TSS variability but the magnitude of TSS variability depended on the variability of the TSS concentration of the sampled region.

In Western Australia, specifically the Pilbara region, the last decade and a half has seen substantial capital dredging projects with the total volume of dredged material in excess of ~70 million m^3^ and the recent Wheatstone gas field project is expected to add another ~45 million m^3^ of dredge spoils to this total [56]. Compliance monitoring of large volume capital dredging and/or frequent maintenance dredging is typically carried out using *in situ* data loggers that measure a range of water quality parameters (TSS concentration, turbidity, light, and sedimentation rate) [57]. In compliance monitoring of dredge operations in Western Australia, it is required of dredging companies to perform environmental impact assessment studies using hydrodynamic modelling of sediment plumes to identify zones of impact and trigger values derived in relation to a water quality parameter and sensitivity to benthic communities [58]. For instance, in the Wheatstone gas field project, a zone of high impact (mortality rate > 50%) was identified along the dredge channels and spoil area. The hydrodynamic model was used to identify trigger values to prompt management responses, with thresholds of TSS > 25 mg L^-1^ for more than 14% of the time, >10 mg L^-1^ for more than 38% of the time, and > 5 mg L^-1^ for more than 63% [58]. The TSS levels set to trigger a management response are monitored using point measurement from the *in situ* data loggers, accepted as providing very accurate and reliable data. However, *in situ* data loggers cannot provide a synoptic view of TSS concentration at reasonable costs over a large spatial extent, which has led environmental managers adopting remote sensing technologies which can provide a synoptic view of plume dynamics and TSS concentration at reasonable costs [59].

Despite the benefits of satellite remote sensing in water quality monitoring, the environmental protection agencies tasked with monitoring the coastal water quality should be aware of potential discrepancies in satellite derived TSS concentration as a result of different satellite sensors and different spatial resolutions. The impact of significant spatial variability in the TSS concentration can affect the results of the satellite derived TSS concentration used in monitoring the water quality. In effect, the monitoring of dredging activity with different satellite-based remote sensing sensors can produce different TSS concentrations even in the same spatial region and depends on which satellite sensor is employed for the compliance monitoring. Thus, this work was carried out to study the variability in TSS concentration at different spatial resolutions in the waters of the Onslow region in northern Western Australia using WV2, Landsat-8 OLI (Operational Land Imager) and MODIS-Aqua data. Specifically, first we tested the capabilities of WV2, Landsat-8 OLI and MODIS-Aqua in resolving the spatial features in areas of sediment plumes caused by dredging activities and river outflows. Second, we quantified the range of TSS concentration variability in the region of the sediment plumes and background waters by degrading the native spatial resolution of each sensor to coarser spatial resolutions. Finally, we discuss the impact of using different spatial resolution sensors in monitoring of water quality as a result of findings from this study.

## Materials and methods

### Study site and context

**Fig 1:**
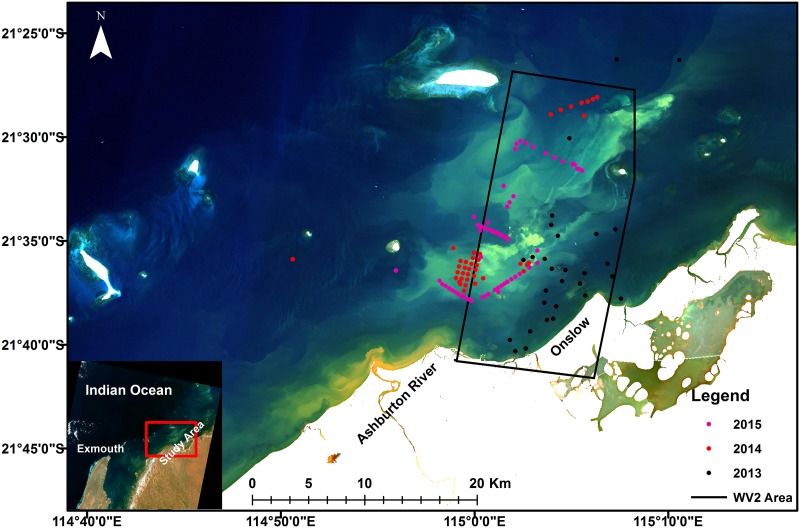
Study site. True color Landsat OLI image showing the locations of field sites in the waters off the coast of Onslow, Western Australia. True color image of the study site is reprinted from Figure 1 in Dorji et al [64] under a Creative Commons Attribution (CC-BY) license (http://creativecommons.org/licenses/by/4.0/). The black polygon added in Fig 1 represents the area where Worldview 2 data were captured on June 13^th^ 2014. The colored dots represent locations of *in situ* data with colors indicating the year of data acquisition.

The study area, the coastal waters of Onslow, fall within the Pilbara region, in Western Australia (see Fig 1). The coastal area of Onslow generally experiences a mean annual temperature of 29.2°C and mean annual rainfall of 296 mm [60]. The study area is generally sheltered from the prevailing south-west winds and sea-swells from the Indian ocean by Barrow Island and the shoals of Lowendal and Montebello Islands, however, the area experiences locally wind-driven waves and seasonal tropical cyclones [61]. The topography of the coastal area generally drives the ebb and flood tides easterly and westerly along the coastline with the flow occasionally disturbed by the locally wind-driven currents. The tides around the shoreline are semi-diurnal with the spring tide ranging from a mean high of 2.5 m to a mean low of 0.6 m [61].

The discovery of the Wheatstone gas field, located at the edge of the continental shelf 200 km off the coast of Onslow (located approximately 1390 km from Perth, Western Australia), has led to the construction of offshore platforms and onshore gas processing plants [61]. The turbidity of the coastal waters of Onslow was previously only affected by natural processes, including seasonal tropical cyclones and episodic river outflows from the Ashburton river which can range the TSS concentration from 15 mg L^-1^ to 5000 mg L^-1^ (with higher TSS concentration closer to the river mouth) with river flow rates of 30 m^3^ s^-1^ to 250 m^3^ s^-1^ [62]. The dredging activity in the near-shore waters of Onslow occurred from May 2013 to December 2015 with an estimated 45 million m^3^ of dredge spoil generated [63]. Such large volumes of dredge spoil are expected to have immediate impact in the immediate area of the dredging and also have some level of impact on the marine habitat in the vicinity of the dredging locations.

### Field remote sensing reflectance and TSS measurements

As part of the Dredging Science Node project 2/3 [65] funded by the Western Australian Marine Science Institution three field campaigns were carried out in October 9^th^– 31^th^, 2013, June 7^th^– 21^st^, 2014 and July 3^rd^– 13^th^ 2015 onboard RV Linnaeus operated by the Commonwealth Scientific and Industrial Research Organization and RV Solander operated by the Australian Institute of Marine Science. The ship-based “Dynamic Above-water radiance (L) and irradiance (E) Collector” (DALEC) [66] was used to measure the remote sensing reflectance (*R*_rs_, in sr^-1^) and *in situ* water sampling methods were used in measuring TSS concentrations. A brief description of the *in situ R*_rs_ measurements using the DALEC and sampling of TSS concentration are provided below. Further details of the *R*_rs_ and TSS concentration data used in this study, including the data collection procedure and data quality control measures, are discussed in depth in Dorji et al. [64].

#### DALEC and TSS data collection and analysis

The DALEC, developed by “In situ Marine Optics”, is an autonomous ship based hyperspectral upwelling radiance (*L*_u_), sky radiance (*L*_sky_) and downwelling irradiance (*E*_d_) collector which takes coincident measurements in 256 spectral bins in the 380 nm to 900 nm spectral range. The *L*_u_, *L*_sky_ and *E*_d_ measurements from the DALEC can be used to compute *R*_rs_ using an ad-hoc *R*_rs_ formulation from Mobley [67] for a uniform sky condition and wind speed less than 5 m s^-1^, as presented in Eq (1).

$$R_{rs}(\lambda )=\frac{L_{\text{u}}(\lambda )\times 0.022L_{\text{sky}}(\lambda )}{E_{d}(\lambda )}$$ (1)

The quality of DALEC data were maintained at two stages. 1) During the data collection stage, we positioned the DALEC instrument at an azimuth angle of ~135° relative to solar direction while the viewing angle of the *L*_u_ and *L*_sky_ sensors were maintained at 40° off nadir and zenith respectively to minimize the sun glint and instrument shading. 2) During the data analysis stage, we visually inspected the *L*_u_ and *L*_sky_ spectra and removed any spectra that were contaminated by sun glint. The remaining spectra free of sun glint were averaged within ± 3 min from TSS sample collection time to generate an average *R*_rs_ spectrum corresponding to that TSS sample.

For *in situ* TSS concentration measurement, we collected a minimum of two 1-liter samples of sea water at a depth of approximately 0.5 m to 1 m at each TSS sample location (see Fig 1). The TSS samples were filtered using Whatman GF/F filters (47 mm diameter, nominal pore size of 0.7 μm) pre-prepared in the laboratory by flushing the filters with 50 mL of deionized water and drying in an oven at 60°C for 24 hrs. The filtered TSS samples were flushed with 50 mL of deionized water to remote salt from the seawater, then dried in the oven at 60°C for 24 hrs and repeatedly measured and dried until consistent measurements were obtained within the tolerance limit of 0.001 mg L^-1^. After performing the quality checks of the *in situ* data there were 48 (*R*_rs_ and TSS) match-up pairs that were selected to establish a TSS algorithm. The range of TSS concentrations used in the algorithm development varied from a low of 2.5 mg L^-1^ to a high of 69.9 mg L^-1^.

### Satellite remote sensing data

#### Satellite data acquisition and atmospheric correction

The satellite data used in this study comprise MODIS-Aqua, Landsat-8 OLI and WV2 acquired around the time when the second field campaign was carried out in June 7^th^–21^st^ 2014. Due to the temporal limitation of the Landsat-8 OLI of 16 days we could not acquire data for all three satellites contemporaneously. However, we acquired three concurrent sets of Landsat OLI and MODIS-Aqua data for May 23^rd^ July 10^th^ and July 26^th^ 2014 that were free of clouds and sun glint. The MODIS-Aqua and WV2 data were acquired for June 13^th^, 2014, which was when the WV2 image was requisitioned over the study region. The spectral bands and the spatial resolutions used in mapping the TSS concentrations were band 1 (620–670 nm) at 250 m, band 4 (640–670 nm) at 30 m and the ‘red band’ (630–690 nm) at 2 m for MODIS-Aqua, Landsat-8 OLI and WV2 respectively.

For this study we used the top of the atmosphere radiance data from MODIS-Aqua available from the NASA LAADS web (http://ladsweb.nascom.nasa.gov/) as geo-located Level 1B data in all 36 spectral bands. All the MODIS-Aqua Level 1B data were atmospherically corrected using the MUMM [49] atmospheric correction as implemented in SeaDAS (version 7.2) [68]. The MUMM atmospheric correction, based on the spatial homogeneity of water leaving radiance and constant aerosol ratios in MODIS 748 nm and 869 nm bands [54], was demonstrated to perform well in the waters over our study region [64].

Radiometrically and geometrically corrected Level 1T Landsat-8 OLI data were obtained from USGS archives using the EarthExplorer (http://earthexplorer.usgs.gov/). The Level 1T Landsat-8 OLI data were atmospherically corrected to marine remote sensing reflectance using the ACOLITE software (available at https://odnature.naturalsciences.be/remsem/software-and-data/acolite) [69]. Two atmospheric correction algorithms are available in ACOLITE, the NIR and SWIR algorithm: the NIR algorithm is based on the selection of the red (655 nm) and NIR (865 nm) bands to account for the aerosol contributions, the SWIR algorithm uses the SWIR1 (1608.5 nm) and SWIR2 (2200.5 nm) bands available on the Landsat-8 OLI sensor. For this study, we selected the SWIR algorithm because it is valid for turbid waters [70], which is the case for our study site where *in situ* TSS concentration was measured as high as 69.6 mg L^-1^ in the vicinity of dredging areas and it is likely higher in the area of the dredge plumes [71]. Further, the SWIR algorithm was shown to be an improvement over the NIR band based atmospheric correction algorithm [69] that was valid for only moderately turbid waters [54, 70].

The WV2 image covered an area of 331 km^2^ over the study area (see Fig 1 for the spatial extent in the study area and the WV2 image). The WV2 data comprise spectral bands in the blue (450–510 nm), green (510–580 nm), red (630–690 nm) and NIR1 (770–895 nm) and are supplied as ortho ready standard WV2 satellite image data at 2 m spatial resolution. The SeaDAS and ACCOLITE platforms were specifically designed and adapted to process, among others, MODIS and Landsat-8 OLI satellite sensor data, but they are not designed to process WV2 data. A study by Martin et al. [72] demonstrated the success of 6S (Second Simulation of a Satellite Signal in the Solar Spectrum) radiative transfer code in the atmospheric correction of satellite data captured in turbid coastal waters. The 6S code predicts the satellite signals at the top of atmosphere between 250–4000 nm based on geometrical conditions, atmospheric models for gaseous components, the aerosol model, spectral conditions, and ground reflectance [73]. Thus, we applied the 6S atmospheric correction method of Kotchenova et al. [74] and obtained the marine surface reflectance using the following input parameters: 1) geometrical conditions were obtained from the solar zenith angle, solar azimuth angle, satellite zenith angle, satellite azimuth angle, image acquisition day and month that was supplied with the WV2 image, 2) the atmospheric model was selected as the Tropical atmospheric model, 3) the aerosol model was selected as the ‘Continental’ aerosol model with visibility of 15 km, 4) The spectral band used was equivalent to the red band of WV2 and ground reflectance was modeled as a homogenous ocean BRDF model with wind speed of 5 m s^-1^, wind azimuth of 220°, salinity of 35 psu and pigment concentration of 0.5 mg/m^3^. The input parameters in 6S were selected to match closely with the conditions over the study region.

#### Validation of atmospheric correction methods

**Fig 2:**
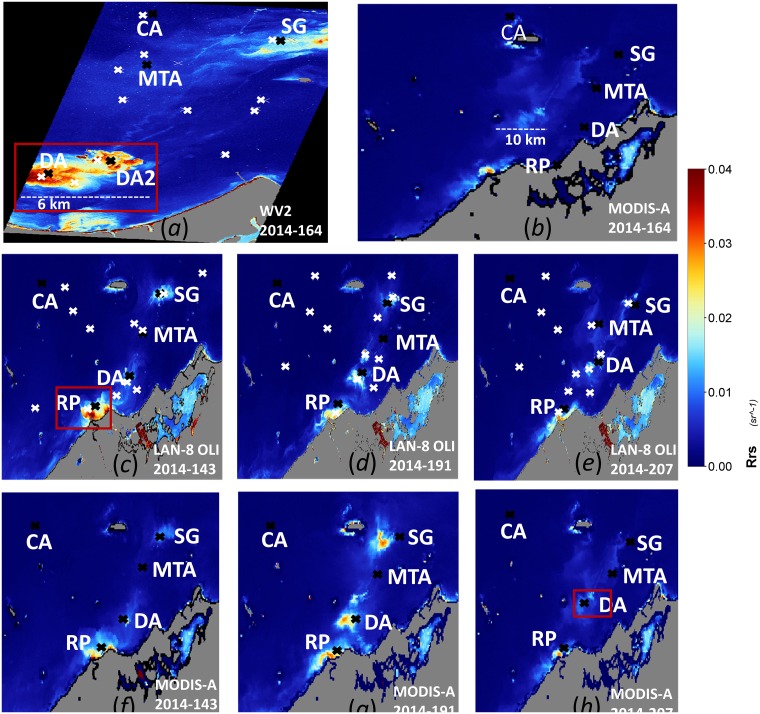
The atmospherically corrected *R*_rs_ (red band) product. (a) and (b) WV2 and MODIS-Aqua on June 13^th^ 2014; (c)-(e) Landsat-8 OLI and (f)-(h) MODIS-Aqua on May 23^rd^, July 10^th^ and July 26^th^ 2014 respectively. The white cross mark on (a), (c)-(e) are the locations of the central pixel of 2.5 km square used in *R*_rs_ product validation. The black cross mark are locations corresponding to Dredged Areas (DA and DA2), Spoil Ground (SG), Clean Area (CA), River Plume (RP) and Moderate Turbid Area (MTA) in each image.

For the *in situ* validation of the atmospheric correction method, only MODIS-Aqua provided concurrent measurements to the DALEC-measured *R*_rs_. The MODIS-Aqua overpass time over the study region on July 13^th^ 2014 was at 06:30 hrs (UTC) while *in situ* TSS and DALEC *R*_rs_ were collected between 02:00–07:30 hrs (UTC). The WV2 and Landsat-8 OLI data were not concurrent with the DALEC-measured *R*_rs_ during any of the Landsat-8 OLI and WV2 overpass times in the study region, thus no *in situ* validation is performed for Landsat-8 OLI and WV2-derived *R*_rs_. The time difference between DALEC *R*_rs_ measurements and MODIS-Aqua overpasses used in the validation was constrained to ±90 min. As a validation of atmospheric correction for Landsat-8 OLI and WV2-derived *R*_rs_, an inter comparison of *R*_rs_ with reference to MODIS-Aqua was performed for the WV2 and Landsat-8 OLI derived *R*_rs_ over the study site for selected locations (see light cross marks in Fig 2a and 2c–2e) representing a range of TSS concentrations. An inter satellite sensor comparison can show significantly different *R*_rs_ values over the same region due to the time difference of data acquisition and the dynamic water conditions where water masses can move and evolve rapidly [54], thus to minimize the effect of satellite data acquisition time difference we used the aggregates of pixel values in a selection of square boxes of 2.5 km in length that represented waters ranging from clear to highly turbid in the image. The length of 2.5 km was chosen because the minimum size of the plumes in the area of study were at least 5 km in length, and the intent was to incorporate pixels within the plumes which are expected to display a small range in *R*_rs_ values. For the MODIS-Aqua and WV2-derived *R*_rs_ comparison, we selected 12 square box regions after visually identifying the areas that ranged in different turbidity from the WV2 image for June 13^th^, 2014 (see white cross marks for central locations of each box in Fig 2a). For the MODIS-Aqua and Landsat-8 OLI derived *R*_rs_ comparison, we selected 12 square boxes per image after visually identifying the areas representing a range of different turbidity levels using Landsat-8 OLI imagery for May 23^rd^, July 10^th^ and July 26^th^, 2014 (see white cross marks in Fig 2c–2e).

#### Degrading the satellite spatial resolution

Quantification of the variability in TSS concentration derived from sensors with different spatial resolutions was assessed by spatially degrading the satellite sensor’s derived TSS products to coarser spatial resolutions than their respective native resolutions of 250 m, 30 m and 2 m for MODIS-Aqua, Landsat-8 OLI and WV2 data respectively. The degradation of the spatial resolution depended on the respective sensor’s native resolution, the MODIS-Aqua TSS data were degraded to 500–5000 m at 500 m intervals, the Landsat-8 OLI TSS data were degraded to 60–4800 m at 60 m intervals, and the WV2 TSS data were degraded to 4–5000 m at 2 m intervals. The spatial resolution was degraded using the aggregate of all available pixel values in a selected region. For example, if MODIS-Aqua 250 m data were to be degraded to 1000 m spatial resolution then all pixels confined within the 1000 m by 1000 m (equivalent to 4 × 4 250 m spatial grids) would be averaged. The locations and size of each selected area were determined visually by assessment of the uniformity of TSS in the region and the spatial resolution of degradation. For each MODIS-Aqua and Landsat-8 OLI TSS image we selected 5 locations, the 1) the center of the dredge area (DA), 2) center of the spoil ground (SG), 3) moderately turbid but spatially uniform area (MTA), 4) clean area (CA) and 5) center of the river plume (RP). For the WV2 TSS image, we also selected 5 locations, but replaced the location of the river plume with the second dredge area (DA2) because the area of the river plume was not covered by the WV2 image (see black cross marks in Fig 2 for the locations).

In addition, the data to visually examine the spatial characteristic of the sediment plumes were generated by spatially degrading the TSS product for all of the study regions from each sensor’s native spatial resolution. The high spatial resolution 2 m WV2 TSS product was degraded to 30 m, 250 m, 500 m, and 1000 m, the spatial resolution of the 30 m Landsat-8 OLI TSS product was degraded to 250 m, 500 m, and 1000 m, and the coarser 250 m spatial resolution of MODIS-Aqua was degraded to 500 m and 1000 m. For the examination of the plume features we focused on the area where the plume was visually evident (see red box in Fig 2a, 2c, and 2h) for the TSS product of June 13^th^ 2014 for WV2, May 23^rd^ for Landsat-8 OLI and July 10^th^ 2014 for MODIS-Aqua.

### Calibration and validation of Multi-Sensor TSS algorithm

The TSS algorithm used in this study is the Semi-Analytic Sediment Model (SASM) from Dorji et al. [64] where the physical form of SASM is based on the principle of radiative transfer and it has been shown that the SASM performs better in the study region compared with simple linear and exponential models. Further, SASM is based on a red spectral band which suits our purpose because all three satellite sensors considered here have red bands which are proven to be effective in mapping TSS concentrations in the turbid region where reflectance does not necessarily co-vary linearly with reflectance. To calibrate the SASM model, the DALEC measured *R*_rs_ was convolved to the respective sensors band’s spectral response functions and then converted to equivalent sub-surface remote sensing reflectance (*r*_rs_). Then all the 48 (*r*_rs_ and TSS) match-up pairs were used in re-calibration of the general form of the SASM in Equation (14) of Dorji et al. [64]. The recalibrated model was validated using the method of Leave-one-out cross-validation (LOOCV) [75] where all but one (*r*_rs_ and TSS) match-up pairs were used in calibration and the remaining one was used in validation until all the match-up pairs were exhausted. The SASM re-calibrated to the respective red bands of MODIS-Aqua, Landsat-8 OLI and WV2 are presented below in Eqs (2), (3) and (4) for MODIS-Aqua in band 1, Landsat-8 OLI in band 4 and WV2 in the red band respectively.

$$TSS=\frac{23.47\times \left(\frac{x}{1-x}\right)}{1-0.69\times \left(\frac{x}{1-x}\right)}$$ (2)

$$TSS=\frac{25.34\times \left(\frac{x}{1-x}\right)}{1-0.69\times \left(\frac{x}{1-x}\right)}$$ (3)

$$TSS=\frac{26.37\times \left(\frac{x}{1-x}\right)}{1-0.69\times \left(\frac{x}{1-x}\right)}$$ (4)

where x=−g1+(g1)2+4gr2rs(λ)2g2, and *r*_*rs*_ (*λ*) = *r*_*rs*_ (band 1) for MODIS-Aqua, *r*_*rs*_ (band 4) for Landsat-8 OLI and *r*_*rs*_ (red band) for WV2, *g*_1_ = 0.084 and *g*_2_ = 0.17

### Mapping of TSS concentration

The *R*_rs_ derived from the atmospherically corrected reflectance of MODIS-Aqua in band 1, Landsat-8 OLI in band 4 and WV2 in the red band for all the corresponding dates of image acquisition were used in mapping the TSS concentration. The respective satellite derived *R*_rs_ were converted to *r*_rs_ using Eq (5) [76] then, the resultant *r*_rs_ was used in the respective satellite sensor’s TSS algorithm given by Eqs (2), (3) and (4) for MODIS-Aqua, Landsat-8 OLI and WV2 respectively.

$$r_{\text{rs}}(\lambda )=\frac{R_{\text{rs}}(\lambda )}{\left(0.52+1.7R_{\text{rs}}(\lambda )\right)}$$ (5)

### Accuracy assessment

The common accuracy assessment methods, Mean Absolute Relative Error (MARE), Absolute Relative Error (ARE) and Root Mean Square Error (RMSE) employed in remote sensing by numerous studies [77–79] were used in this study to compare model-derived and ‘true’ *R*_rs_ and TSS values. In this study we refer to ‘true’ value as the *in situ* measurements or MODIS-Aqua derived *R*_rs_ or TSS values. We also considered the correlation coefficient (*r*) defined in Eq (9), although *r* cannot be strictly used in assessing the accuracy between two models because a high *r* value does not necessarily mean a better prediction because the systematic model error can also lead to over and/or under prediction [79]. We used *r* to gauge the presence of positive correlation between the models. RMSE, as defined in Eq (8), is the most common accuracy assessment used to indicate average error of a model. Because of its susceptibility to outliers we resorted to using RMSE to evaluate *in situ* validation of TSS algorithms only, where in the *in situ* model validation the model-derived TSS concentration is not expected to deviate significantly from the *in situ* TSS measurements. However, in the accuracy assessment of TSS concentration derived from the satellite images, the TSS concentration can be highly variable and possibly include outliers, which can limit the use of RMSE accuracy assessment in such cases. Thus, the MARE and ARE as defined in Eqs (6) and (7) respectively, were deemed more appropriate for satellite image derived TSS comparison. Further, the MARE and ARE are scale independent and provide errors in percentages, which better facilitates the comparative study of TSS concentrations produced by different satellite sensors. Thus, accuracy assessment for quantitative comparison of TSS concentration derived from different sensors was performed using MARE and ARE. However, it should be noted that negative or zero, model-derived or ‘true’ values can result an unreliable accuracy estimates in MARE and ARE calculation. In this study, in the atmospheric correction process of the satellite images, the *R*_rs_ values were tested for negative or zero values to be flagged as ‘bad’ pixels and removed from subsequent analysis.

$$\text{MARE}=\frac{\sum_{i=1}^{n}\left|\left(x_{i}-y_{i}\right)/y_{i}\right|}{n}\times 100\%$$ (6)

$$\text{ARE}=\frac{\left|x_{i}-y_{i}\right|}{y_{i}}\times 100\%$$ (7)

$$\text{RMSE}=\sqrt{\frac{\sum_{i=1}^{n}{\left(x_{i}-y_{i}\right)}^{2}}{n}}$$ (8)

$$r=\frac{n\sum x_{i}y_{i}\;\sum x_{i}\sum y_{i}}{\sqrt{n\sum x_{i}{}^{2}\;(\sum x_{i}{)}^{2}}\sqrt{n\sum y_{i}{}^{2}\;(\sum y_{i}{)}^{2}}}$$ (9)

where *n* is the total number of samples, *x*_*i*_ is the model-derived TSS and *y*_*i*_ is the ‘true’ TSS.

## Results

### Validation of TSS algorithms

**Fig 3:**
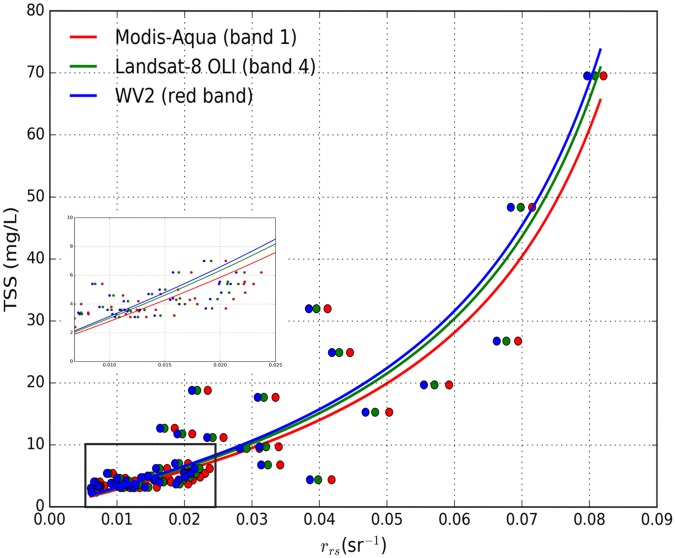
The TSS model curves for MODIS-Aqua (blue), Landsat-8 OLI (green) and WV2 (red). The *in situ* data points are shown by filled circles with the same colour profile as respective TSS model curves. The data for TSS < 10 mg L^-1^ and *r*_rs_ < 0.025 sr^-1^ are also shown in the blow out version of the plot.

The result from the LOOCV method used in calibration and validation of the TSS algorithms in Eqs (2), (3) and (4) are presented in Table 1. Further, the corresponding TSS model curves for MODIS-Aqua in band 1, Landsat-8 OLI in band 4 and WV2 in the red band are shown in Fig 3. The results from all three sensor’s TSS algorithms produce similar results in terms of MARE, RMSE and *r* values. The similar results between all three TSS algorithm’s validation are expected because all three algorithms use the respective sensor’s red band with slight variation in spectral response function of each sensor.

**Table 1:** Validation results for MODIS-Aqua, Landsat-8 OLI, and WV2 TSS algorithms.

SASM Model	MARE (%)	RMSE (mg L^-1^)	*r*
Modis-Aqua	33.33	5.75	0.89
Landsat-8 OLI	33.36	5.73	0.89
WorldView 2	33.34	5.68	0.89

### Multi-Sensor atmospheric correction validations

Validation of the atmospheric correction methods for the different satellite sensors involved two methods: 1) *in situ* validation for MODIS-Aqua atmospheric correction methods and 2) inter-sensor *R*_rs_ validation between MODIS-Aqua, WV2 and Landsat-8 OLI. In both the validation methods, type-II linear regression from [80] was used because type-I regression typically assumes the dependent (‘true’) variable is error free, but this is not the case even in *in situ* measurements [81].

**Fig 4:**
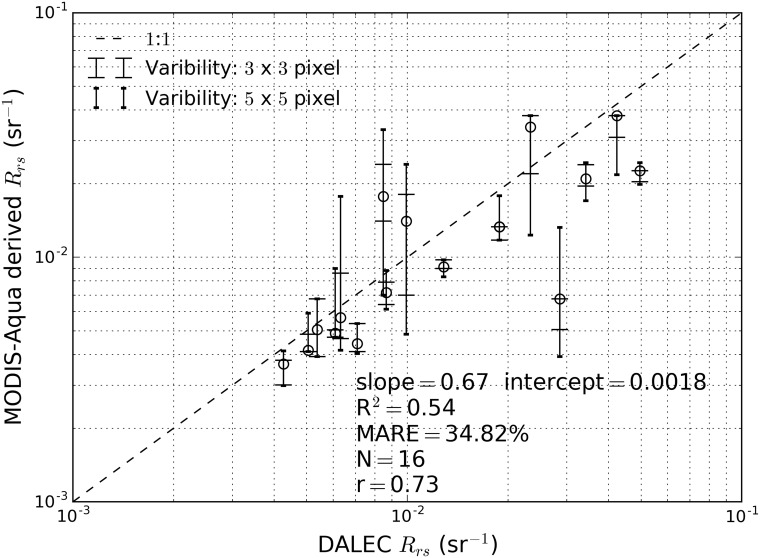
In situ validation of DALEC-measured *R*_rs_ and MODIS-Aqua derived *R*_rs_ for match-up data within ± 90 min from the satellite overpass. The error bars indicate the maximum and minimum MODIS *R*_rs_ values in 3 × 3 and 5 × 5 pixel extents.

The validation result for *in situ* atmospheric correction for MODIS-Aqua using the DALEC-derived *R*_rs_ is shown in Fig 4. The error bars on the data points in Fig 4 indicate the minimum and maximum values of *R*_rs_ within 3 × 3 and 5 × 5 pixel extents. In Fig 4 we observe that the majority of the data points were within the 1:1 line considering the error bars from *R*_rs_ variability in a 5 by 5-pixel window. However, there are also a few data points whose error bars do not overlap with the 1:1 line and resulted in ARE as high as 109.64% between the *in situ* DALEC *R*_rs_ measurement and MODIS-Aqua derived *R*_rs_. The overall MARE of all data points was 34.82% with slope of 0.67, intercept of 0.0018 and R^2^ of 0.54 as obtained from Type-II regression. Additional observation we can make from Fig 4 are that as the pixel window increases from a 3 × 3 to a 5 × 5 pixel window, the upper and lower error bounds also increase, showing that the water is highly variable in *R*_rs_ values. This spatial variability in *R*_rs_ is associated with the spatial variability in TSS.

**Fig 5:**
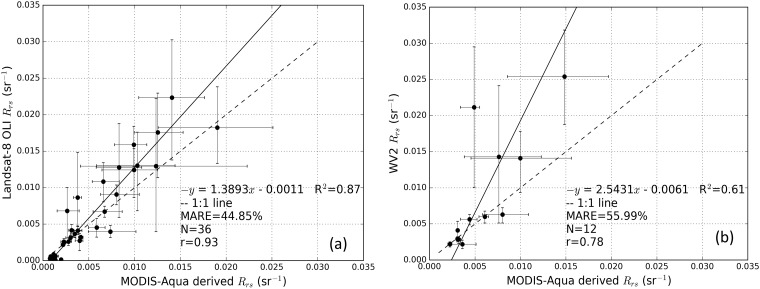
Inter-satellite *R*_rs_ product validation results. (a) 2014 MODIS-Aqua vs Landsat-8 OLI *R*_rs_ product validation from May 23^rd^, July 10^th^ and July 26^th^ 2014; (b) MODIS-Aqua vs WV2 *R*_rs_ product validation for *R*_rs_ from June 13^th^. The error bars indicate the 17.5 percentile (lower limit) and 82.5 percentile (upper limit) of pixel values from a 2.5 km width box for each respective satellite sensors derived *R*_rs_. Dashed lines indicate the 1: 1 relationship.

The result of the inter-sensor validation of the *R*_rs_ product is shown in Fig 5a and 5b. From Fig 5a and 5b we observe that the inter-sensor *R*_rs_ product validation of MODIS-Aqua vs Landsat-8 OLI (Fig 5a) with MARE of 44.85% showed a better result than MODIS-Aqua vs WV2 (Fig 5b) with a MARE of 55.99%. In addition, the ARE results in Fig 5a were also better with the smallest ARE and largest ARE of 0.15% and 158.11% while in Fig 5b the smallest ARE and largest ARE were 1.20% and 332% respectively. Further, in Fig 5a the type-II linear regression indicates that there is high correlation, with R^2^ = 0.87, between MODIS-Aqua and Landsat-8 OLI derived *R*_rs_, with most data points falling along the 1:1 line, considering the *R*_rs_ variability within a 2.5 km width square box (indicated by error bars in Fig 5a and 5b with the 17.5 and 82.5 percentile *R*_rs_ values). The correlation between MODIS-Aqua and WV2, as shown in Fig 5b, was lower, with R^2^ = 0.61 with some data points failing to fall within the 1:1 line even after considering the errors from *R*_rs_ variability in the 2.5 km square box. However, the majority of the data points in both Fig 5a and 5b show that MODIS-derived *R*_rs_ are lower than either WV2 or Landsat-8 OLI derived *R*_rs_ for *R*_rs_ > 0.005 sr^-1^.

### Sediment plume features examination

**Fig 6:**
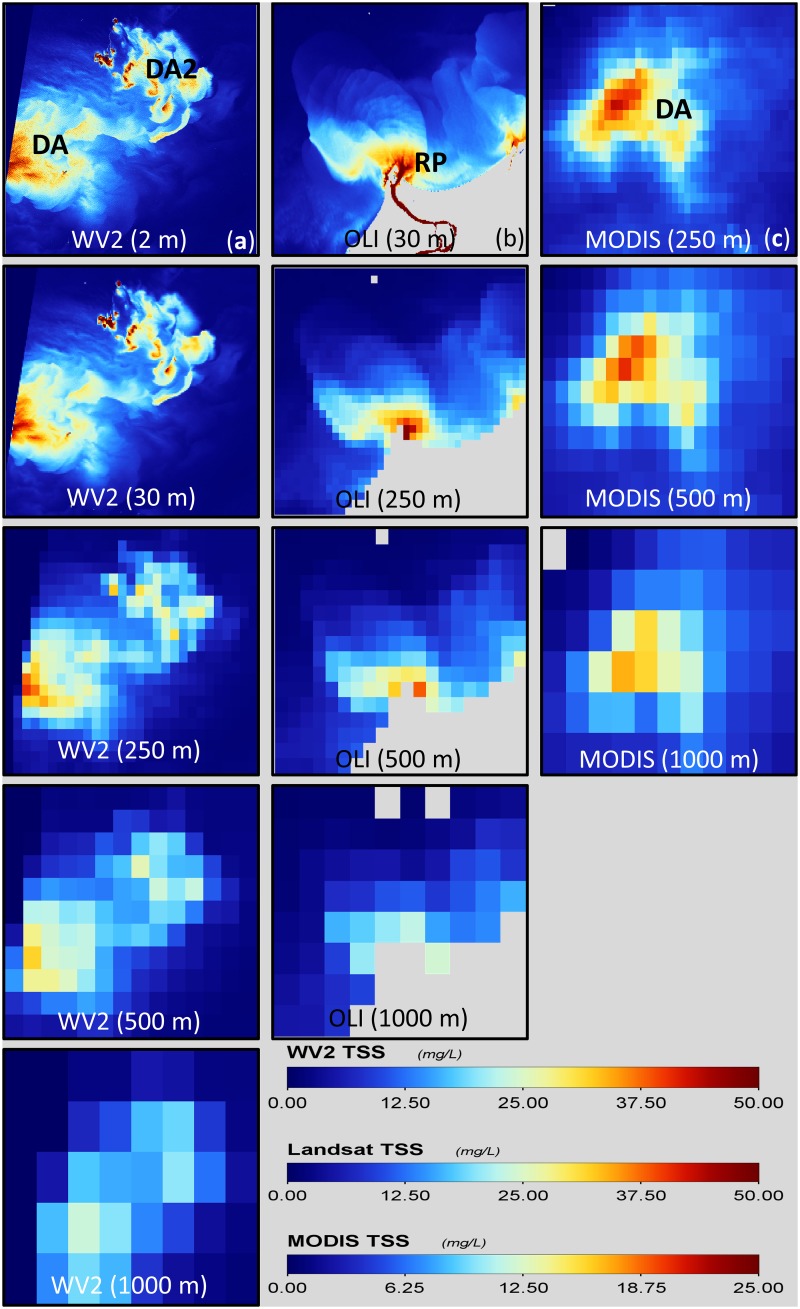
Spatially degraded images of the Dredge Area (DA) and River Plume (RP). Extracted from images in Fig 2a, 2d and 2i corresponding to (a) WV2, (b) Landsat-8 OLI and (c) MODIS-Aqua.

Few selected regions within the study sites in Fig 1 (shown by red boxes in Fig 2a, 2c and 2h) which are spatially degraded to lower spatial resolutions are shown in Fig 6a–6c for WV2, Landsat-8 OLI and MODIS-Aqua sensors respectively. Subsequent images from the top row to bottom row in Fig 6a–6c are spatially degraded to a coarser spatial resolution. In Fig 6a, showing WV2 at 2 m spatial resolution, we are able to visually identify even the fine spatial features in the sediment plumes adjacent to the large turbidity features which are very evident. Similar spatial features as those observed at 2 m spatial resolution are still evident in the degraded lower spatial resolution of 30 m. As the spatial resolution is degraded to 250 m and 500 m the fine spatial features which were evident at 2 m and 30 m spatial resolution are no longer visible, but we can still identify the two large distinct plume regions (DA and DA2 in Fig 6a) which are visible enough to be distinguished as two separate regions of plume when compared with the surrounding areas in DA and DA2. In the lowest spatial resolution of 1000 m, we can no longer clearly discern even the two distinct DA and DA2 plumes observed at the 250 m and 500 m spatial resolutions. The separate regions of DA and DA2 are fused together to appear as one large region of turbid plume when compared with the surrounding background data. In Fig 6b, showing Landsat-8 OLI data at 30 m spatial resolution, we can distinguish the fine features of the river plume, but as the spatial resolution is degraded to 250 m, 500 m and 1000 m only the larger boundaries of the sediment plumes remain visible as the finer features are replaced by the coarser grids at degraded spatial resolutions. Similarly, in Fig 6c showing MODIS-Aqua data, we can clearly observe the dredge plume in the 250 m and 500 m spatial resolution images, but the 1000 m spatial resolution image loses the details that are observed at the higher spatial resolutions.

### Quantification of TSS in sediment plumes

**Fig 7:**
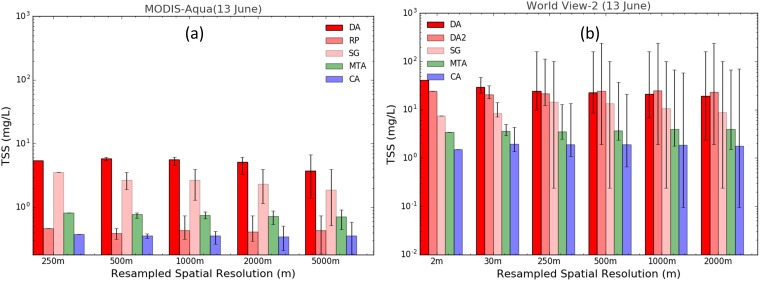
Average TSS concentration. (a) MODIS-Aqua and (b) WV2 at their respective native and degraded spatial resolutions, averaged over the areas: dredge plume (DA and DA2), Spoil Ground (SG), River Plume (RP), Moderate Turbid Area (MTA) and Clean Area (CA). The error bars indicate the minimum and maximum TSS concentrations in each spatial grid.

Fig 7a and 7b show the histograms of TSS concentrations derived from pixels located within the clean area (CA) which represents the background water to the turbid dredged area (DA) for all the images at MODIS-Aqua and WV2 sensor’s native spatial resolution as well as spatially degraded resolutions respectively. The degraded resolutions encompass more pixels and the histogram shows the average TSS value of each area, with the error bars indicating the maximum and minimum TSS values of the native resolution pixels within each area. The comparative results between MODIS-Aqua and WV2 for the June 13^th^ 2014 show that MODIS-Aqua derived average TSS values are relatively lower than WV2 derived average TSS for the regions DA, RP, SG and MTA. At sensor native resolution (2 m for WV2 and 250 m for MODIS-Aqua) the MODIS-Aqua derived TSS for the turbid regions (DA, SG and RP) were ~8.5 times less than WV2 derived TSS concentrations. In terms of average TSS derived at different spatial resolutions for the WV2 image (Fig 7b) we observe that in the plume/turbid areas (DA), the average TSS concentration decreased as the spatial resolution became coarser and the MARE between average TSS derived from 2 m and 2000 m spatial resolution in DA was 114.46%. Likewise, a similar trend was observed for the MODIS-Aqua images, with the MARE between 250 m and 5000 m spatial resolutions of 30.80% for MODIS-Aqua.

The variability in TSS concentration in different regions is represented by error bars (minimum and maximum TSS concentration in each spatial grid) in Fig 7a and 7b. The error bars in Fig 7a and 7b show that for all regions considered, the range of TSS variability increases as spatial resolution gets coarser and the area encompassed increases. In the region of the dredge plume (DA) the TSS concentration ranged from a low of 2.3 mg L^-1^ to a high of 160 mg L^-1^ for the WV2 image at the spatial resolution of 2000 m while for MODIS-Aqua, at a spatial resolution of 5000 m, by contrast only displayed TSS in the range of 1.4 mg L^-1^ to 6.6 mg L^-1^.

**Fig 8:**
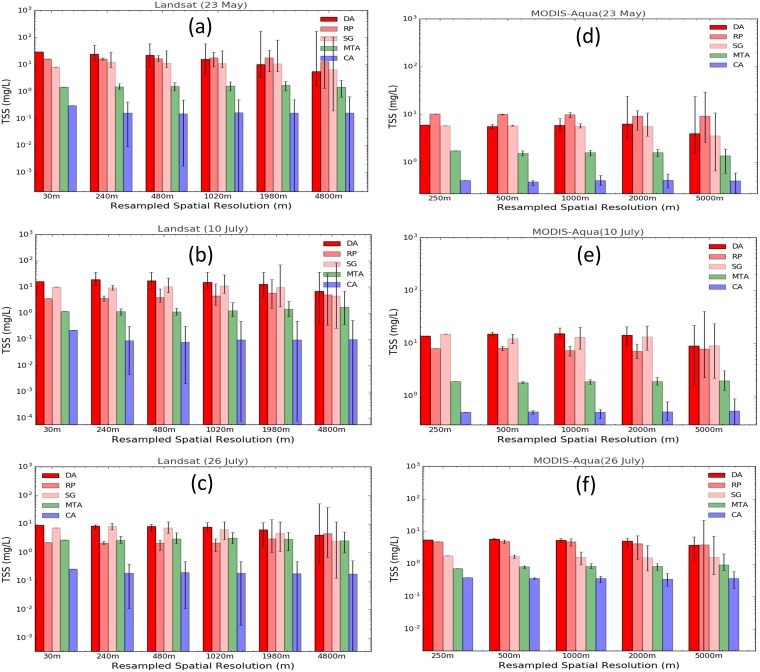
Average TSS concentration. (a)—(c) Landsat-8 OLI and (d)—(f) MODIS-Aqua at their respective native and degraded spatial resolutions in the dredge plume (DA), Spoil Ground (SG), River Plume (RP), Moderate Turbid Area (MTA) and Clean Area (CA). The error bars indicate the minimum and maximum TSS concentrations in each spatial grid.

Fig 8a–8f show histograms of the TSS concentration derived using Landsat-8 OLI and MODIS-Aqua data for May 23^rd^, July 10^th^ and July 26^th^ 2014 for regions DA, CA, MTA, RP and SG at native and degraded spatial resolutions. For all three dates, the TSS concentration derived using Landsat-8 OLI images were higher than the MODIS-Aqua for the turbid (DA, SG and RP) and moderately turbid (MTA) regions while the MODIS-Aqua derived TSS was higher than the Landsat-8 OLI for the clean area (CA). For the turbid regions (DA, RP and SG) the ARE between MODIS-Aqua and Landsat-8 OLI derived TSS ranged from 2.3% to 304.68% with higher ARE at the higher spatial resolution for all Landsat-8 OLI and MODIS-Aqua image pairs. For the regions of moderately turbid (MTA) and clean area (CA) the ARE in TSS concentration ranged from 44.22% to 82.08% with a maximum of 4% variability in ARE for all different spatial resolutions within any Landsat-8 OLI and MODIS-Aqua image pair.

In general, apart from the MODIS-Aqua image of the May 23^rd^ 2014 (Fig 8d) all TSS concentrations derived for turbid regions (DA, SG and RP) show general trends in which the mean TSS concentrations of the coarser spatial resolution grid are lower than the mean TSS concentrations derived at higher spatial resolution. Further, the variability in TSS concentration as represented by minimum and maximum TSS concentrations in the spatial grid increases as the spatial grids get coarser and cover a larger extent. The range of TSS concentration as derived by Landsat-8 OLI and MODIS-Aqua varied from 5.59 mg L^-1^ to 29.15 mg L^-1^ and 3.9 mg L^-1^ to 6.31 mg L^-1^ in the turbid regions (DA, SG and RP) respectively while the TSS concentration ranged from 0.38 mg L^-1^ to 0.43 mg L^-1^ for MODIS-Aqua and 0.14 mg L^-1^ to 0.30 mg L^-1^ for Landsat-8 OLI in the background waters (CA).

## Discussion

### Data and methodological limitations

The results presented demonstrate the differences observed in remotely sensed TSS concentrations for three different sensors and for varying spatial scales of monitoring. The remote sensing instruments, WV2, MODIS-Aqua and Landsat-8 OLI considered in this study have their own radiometric characteristics and atmospheric correction methods that are best suited to each individual sensor. Apart from the radiometric and atmospheric correction methodologies considered for each sensor we also have to take into account the different image acquisition times when attempting to compare the results of the different sensors. The miss-match between the different sensor image acquisition times leads to the situation where the water mass, or the feature of interest such as a sediment plume, may move and alter in spatial distribution, thus the comparison of the *R*_rs_ was based on a spatial subset of pixels contained within a square region of length 2.5 km, the average distance the surface current for June 13^th^ 2014 in the study region was estimated to move a water mass within the acquisition time differences (P. Branson, personal communication, July 4^th^ 2016). Further, the effect of pixel resolution and the size of the spatial domain on the TSS product was studied by degrading the spatial resolution of the TSS products for each sensor to coarser and larger grids.

The inter-sensor TSS algorithms considered in this study were all calibrated using the same *in situ* TSS and *R*_rs_ measurements in the red bands of the respective sensors and the *in situ* validation result of the TSS algorithms for all three satellite sensors were within MARE of 33.33% to 33.36%. Fig 3 shows the close similarity in the algorithm curves for each sensor, with a maximum relative difference of ~10% between TSS values occurring at higher *R*_rs_. However, comparison between TSS algorithm curves should take into account the differences in the spectral response of each sensor, thus the *R*_rs_ value for the same body of water would be expected to be slightly different for each sensor, as indicated by the horizontal displacement of the individual data points in Fig 3. Nonetheless, the value of 10% is a reasonable estimate of the upper limit of the differences in TSS to be expected simply due to differences between sensor algorithms. The use of different atmospheric correction methods for different sensors can cause discrepancies in the final derived TSS products, thus it is vital to account for such discrepancies in atmospheric correction methods. The *R*_rs_ results for the MODIS-Aqua which were validated using the *in situ R*_rs_ data showed that MODIS-Aqua had MARE of 33.82%. The WV2 and Landsat-8 OLI atmospheric correction results which were “validated” against the MODIS-Aqua *R*_rs_ data had MARE of 55.99% for WV2 vs. MODIS-Aqua, and 44.85% for MODIS-Aqua vs. Landsat-8 OLI. The high MARE values of inter sensor validation may be expected because of the acquisition time differences between satellite sensor data that were in excess of 3 hrs between MODIS-Aqua and WV2, and 3.75 hrs between MODIS-Aqua and Landsat-8 OLI. In areas of the turbid dredged plumes (DA and DA2 in Fig 2a) the MODIS-Aqua *R*_rs_ derived using the MUMM atmospheric correction method is particularly low when compared with *R*_rs_ derived from WV2 using the 6S atmospheric correction method. The highest ARE were between the *R*_rs_ derived from the MUMM and 6S atmospheric correction methods at 332% while the lowest ARE of 1.2% were observed in the region of the background waters (CA in Fig 2a). The underestimation of *R*_rs_ by the MUMM atmospheric correction method could be because it was designed for moderately turbid waters [69] and fails to retrieve *R*_rs_ correctly in highly turbid waters of the dredge plumes. Similar under estimation of *R*_rs_ in the turbid region (DA in Fig 2c–2h) by the MUMM atmospheric correction method applied to the MODIS-Aqua image was observed when compared with *R*_rs_ derived from the SWIR atmospheric correction applied to the Landsat-8 OLI which was adapted for the turbid waters [70].

### General observation and recommendations

The effect of different spatial resolutions of the satellite sensors on identifying and mapping the fine features in the dredge plumes are evident from the results. The higher spatial resolution satellite sensors, no doubt, have the benefit of identifying even the fine features in the sediment plumes. For the size and scale of images displayed, the 30 m Landsat-8 OLI and 2 m WV2 TSS products shows similarly fine features, but as the spatial resolution is degraded to larger pixel sizes the fine features are no longer visible, as seen in images with the spatial resolution greater than 250 m (see Fig 6). The fine details observed with the high spatial resolutions of WV2 at 2 m and Landsat-8 OLI and 30 m native spatial resolution makes these two sensors capable of resolving fine spatial details in the surface turbidity features and shows the capability of their application in spatial features/extent mapping of the sediment plumes when compared with MODIS-Aqua sensors. From the perspective of dredge plume monitoring for environmental impact assessment or compliance, the finer details available in the higher resolution satellite data provide better resolution of the spatial extent of dredge plumes, and this in turn translates to a higher confidence in the product. For instance, the extent of the dredge plume in the lateral direction when measured with the high resolution WV2 image was ~6 km, while the MODIS-Aqua derived measurement was ~10 km. However, marine and environmental protection agencies should carefully weigh the cost and benefit of using different spatial resolution sensors. Both the WV2 and Landsat-8 OLI data are able to identify the fine features of the dredge plume, but users should be mindful that the WV2 data are not freely accessible, as is Landsat-8 OLI. Further, if the requirement of the agencies were just to map the extent of dredge plume then MODIS 250 m spatial resolution shows similar capability in mapping the larger TSS spatial features, but not the fine features and details as seen in the high resolution WV2 and Landsat-8 OLI images.

The general trend observed in quantified TSS concentration (Figs 7 and 8) is that as the spatial resolution gets coarser and the spatial extent increases the mean TSS concentration decreases for all three different sensors for turbid regions (DA, SG and RP) while the mean TSS concentrations for CA and MTA remain relatively uniform. Depending on the spatial resolution, the mean TSS concentration results for different spatial resolutions by the same satellite sensors are different and it is shown to decrease as spatial resolution gets coarser for turbid regions. The decrease in TSS concentration with coarser spatial resolutions are observed because of the inclusion of background and lower turbid waters in averaging as the spatial grids get larger. In monitoring of TSS concentration in turbid regions it is important for environmental agencies to be mindful of the result from this study where it shows the effect of the coarser spatial resolution sensors in inclusion of background and lower TSS concentration neighboring pixels producing a lower average TSS concentration than the TSS concentration of the sediment plume over a small spatial extent, particularly when the size of the sediment plume is smaller than the spatial resolution of the satellite sensor. However, our results did not show that such an effect is observed in regions where the turbidity is uniformly distributed over a relatively large spatial extent.

The quantification of TSS concentrations variability results (see S1 Text for details) show that in the background, CA (see S3 Fig), spatially uniform and moderately turbid waters, MTA (see S2 Fig), the TSS variability remains similar across different spatial resolutions for each sensor. The TSS variability across different spatial resolutions (250 m– 2000 m) for CA and MTA were mostly below ~5% from the mean TSS concentrations of the respective region, with the exception of Landsat-8 OLI in MTA which had TSS variability of 10.39%. The low TSS variation is expected in the CA and MTA regions because the CA, which is approximately 30 km from the dredge region, is expected to remain undisturbed by the dredging activities and has a natural background level of TSS concentration without disturbance from anthropogenic processes. Further, the MTA region, which has spatially uniform TSS concentration, is expected to show minimum variance when spatially degraded to represent coarser spatial resolution. However, in the turbid regions (DA, SG and RP) the TSS variability was higher, with 16.96%, 54.09%, and 12.05% for MODIS-Aqua, Landsat-8 OLI and WV2 respectively. The higher TSS variability in the turbid regions, the regions of dredge and river plumes, can be associated with higher TSS gradient in each region. The mean TSS concentration derived by different satellite sensors was also different for each sensor.

The MODIS-Aqua sensor produced mean TSS concentrations of 12.67±2.15 mg L^-1^, 1.89±0.04 mg L^-1^, and 0.51±0.02 mg L^-1^ for the DA, MTA and CA regions respectively. Likewise, for Landsat-8 OLI and WV2 sensors, the mean TSS concentrations in the DA, MTA and CA regions were quantified to be 11.34±6.13 mg L^-1^, 1.61±0.07 mg L^-1^, and 0.16±0.02 mg L^-1^ for Landsat-8 OLI and 22.04.34±2.65 mg L^-1^, 3.85±0.19 mg L^-1^, and 1.84±0.06 mg L^-1^ for WV2. Thus, in monitoring TSS concentration, it should be noted that the TSS variability observed by the satellite sensors is not only associated with the different satellite sensor’s spatial resolution, but also the horizontal spatial distribution of TSS as well.

## Conclusion

The aim of this study was to highlight the effect of the sensor spatial resolution on quantification of TSS concentration in turbid sediment plumes. Results from this study show that different satellite sensors with different spatial resolutions can produce different TSS concentrations, particularly in regions of spatially variable TSS. The WV2 sensor, with 2 m spatial resolution, was shown to generate TSS concentrations as high as 160 mg L^-1^ in the region of the dredge plumes while the highest TSS concentration generated by MODIS-Aqua with 250 m spatial resolution was 23.6 mg L^-1^. Even for the same satellite sensor degraded to different spatial resolutions, the TSS concentrations in the non-uniform turbid regions varied by 114.46%, 304.68% and 38.2% for WV2, Landsat-8 OLI and MODIS-Aqua respectively as the sensor resolution was degraded and the spatial extent increased. In the region of background water and uniformly turbid waters, the mean TSS concentration was observed to be uniform as the sensor resolution was degraded and the spatial extent was increased. Thus, in the context of TSS monitoring of the coastal waters, and particularly for environmental compliance monitoring for dredge operations, users must be mindful of the fact that different satellite sensors produce different TSS concentrations with higher spatial resolution satellite sensors reporting higher TSS values. Also, higher spatial resolution sensors are able to resolve fine turbidity features while lower spatial resolution sensors are only able to resolve the larger spatial extent of the sediment plumes.

## Supporting information

S1 Fig TSS concentration variability at different spatial resolution derived from MODIS-Aqua, WV2 and Landsat-8 OLI in Dredge Area (DA). (TIF)

S2 Fig TSS concentration variability at different spatial resolution derived from MODIS-Aqua, WV2 and Landsat-8 OLI in Moderate Turbid Area (MTA). (TIF)

S3 Fig TSS concentration variability at different spatial resolution derived from MODIS-Aqua, WV2 and Landsat-8 OLI in Clean Area (CA). (TIF)

S1 Text Inter-sensor TSS variability results in dredge, moderate turbid, and clean area. (DOCX)
