# Supplementary figures and images for: Impact of the spatial resolution of satellite remote sensing sensors in the quantification of total suspended sediment concentration: A case study in turbid waters of Northern Western Australia
Source: PLoS One. 2017 Apr 5;12(4):e0175042. doi: 10.1371/journal.pone.0175042 (PMC5381897; doi:10.1371/journal.pone.0175042)

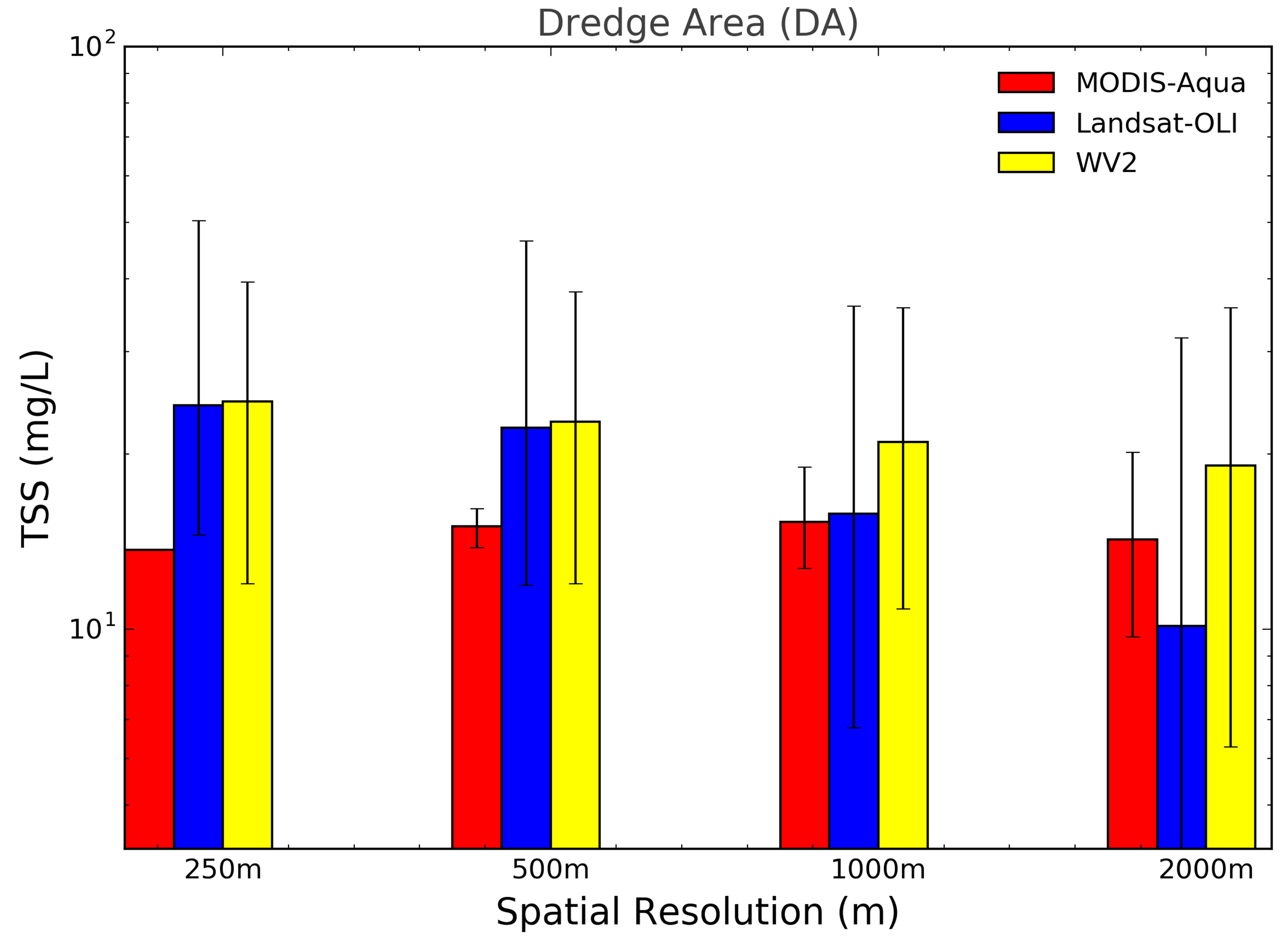

Supplement: S1 Fig — (TIF) [file pone.0175042.s001.tif]

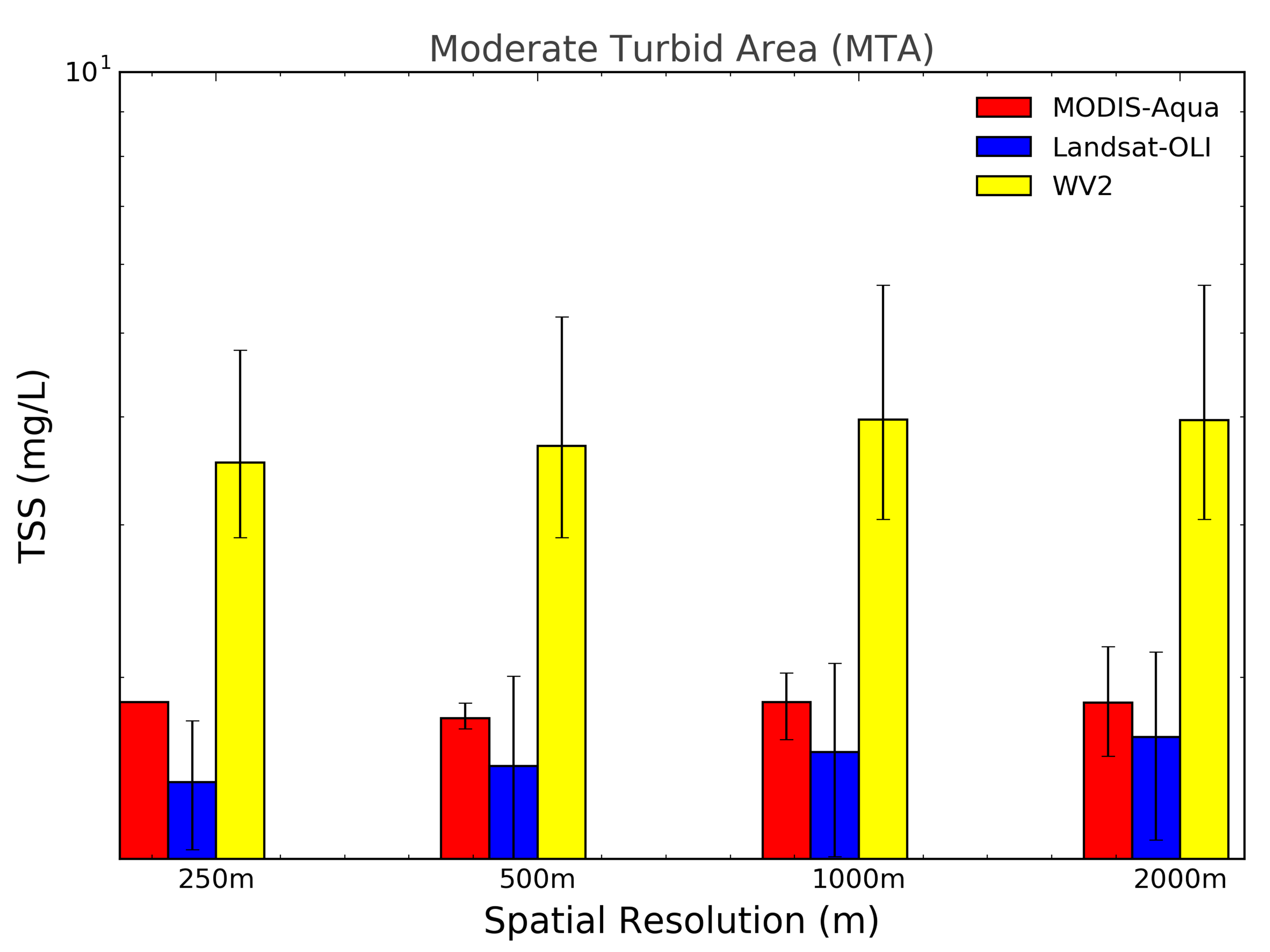

Supplement: S2 Fig — (TIF) [file pone.0175042.s002.tif]

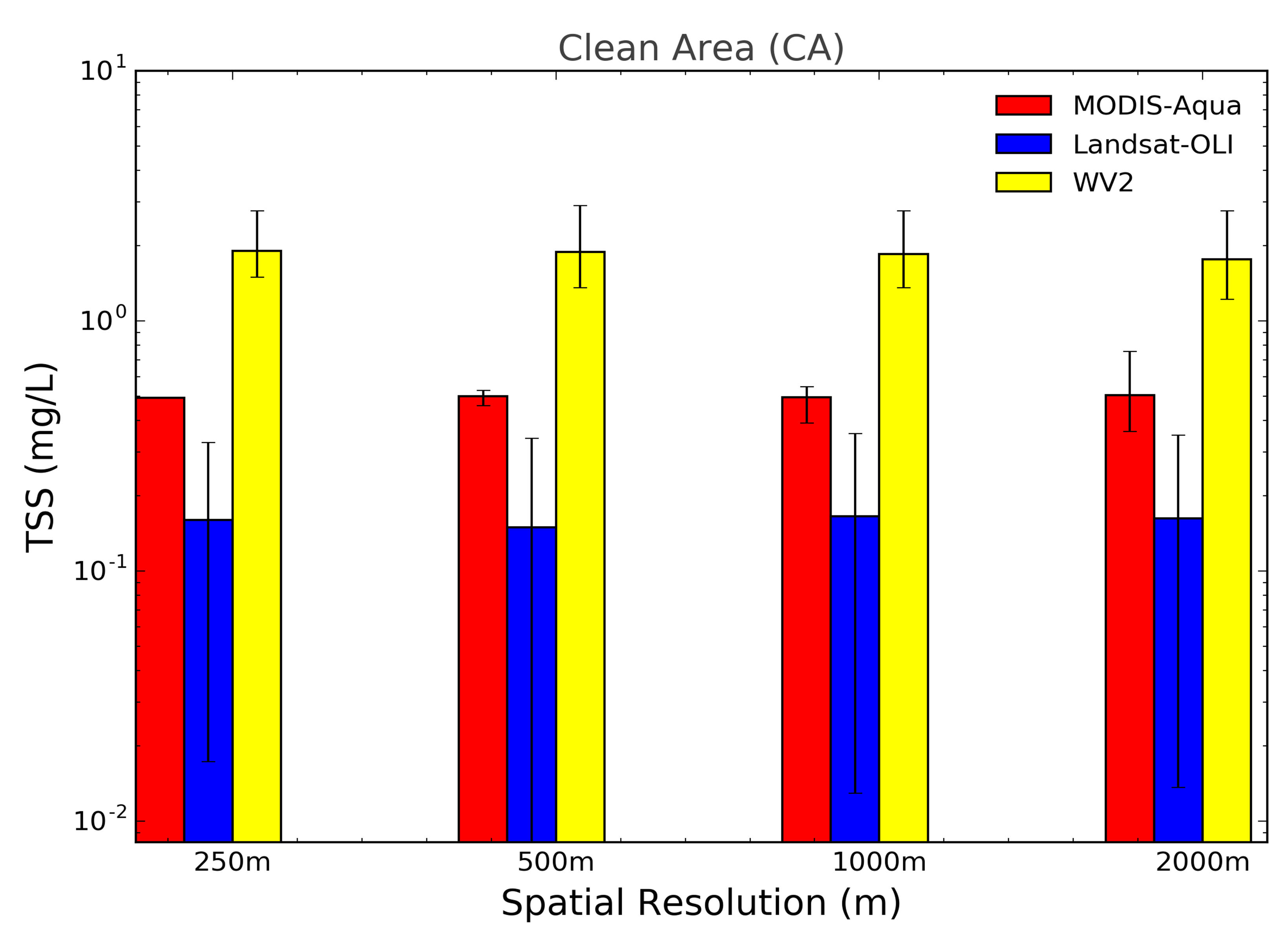

Supplement: S3 Fig — (TIF) [file pone.0175042.s003.tif]
