# Supplementary material for: Impact of the spatial resolution of satellite remote sensing sensors in the quantification of total suspended sediment concentration: A case study in turbid waters of Northern Western Australia
Source: PLoS One. 2017 Apr 5;12(4):e0175042. doi: 10.1371/journal.pone.0175042 (PMC5381897; doi:10.1371/journal.pone.0175042)
Supplement: S1 Text — (DOCX) [file pone.0175042.s004.docx]

Supplementary Information

Inter-Sensor TSS Variability in Dredge, Moderate Turbid, and Clear Area

S1-S3 Figs shows the variability in TSS concentration at 250 m, 500 m, 1000 m, and 2000 m spatial resolutions for MODIS-Aqua, WV2 and Landsat-8 OLI in the DA, MTA and CA respectively. From S1 – S3 Figs, we observe that in the MTA and CA (see S2 and S3 Fig), TSS concentrations derived by each individual sensor remained similar across different spatial resolutions. In the DA (S1 Fig) the MODIS-Aqua derived TSS concentration at 250 m spatial resolution is lower than the Landsat-8 OLI and WV2 sensor, but as the spatial resolution gets coarser all sensors’ derived TSS concentrations are similar within the respective error bars. In the turbid region (S1 Fig) the MODIS-Aqua sensors displayed little variability as the resolutions were decreased and the spatial extent was increased, but Landsat-OLI and WV2 both displayed a decrease in the mean TSS values. However, S2 and S3 Figs shows that the TSS concentrations derived by the individual sensors were similar across different spatial resolutions, but overall the WV2-derived TSS concentration remained high and Landsat-8 OLI-derived TSS concentration remained low across all spatial resolutions. In S1-S3 Figs, across different spatial resolution grids (250 m – 2000 m), the DA, MTA and CA region of the study site was quantified to have mean TSS concentration of 12.67±2.15 mg L^-1^, 1.89±0.04 mg L^-1^, and 0.51±0.02 mg L^-1^ respectively for MODIS-Aqua sensor. Likewise, for Landsat-8 OLI and WV2 sensors mean TSS concentration in DA, MTA and CA regions were quantified to be 11.34±6.13 mg L^-1^, 1.61±0.07 mg L^-1^, and 0.16±0.02 mg L^-1^ and be 22.04.34±2.65 mg L^-1^, 3.85±0.19 mg L^-1^, and 1.84±0.06 mg L^-1^ respectively. Further, the range of TSS concentration observed were in CA were 0.70 mg L^-1^ to 4.02 mg L^-1^, 0.05 mg L^-1^ to 3.2 mg L^-1^, and 0.35 mg L^-1^ to 1.9 mg L^-1^ for WV2, Landsat-8 OLI and MODIS-Aqua sensors respectively. Likewise, in the turbid regions (DA, SG and RP) the TSS concentrations varied in the ranges of 19.12 mg L^-1^ to 41.02 mg L^-1^, 4.06 mg L^-1^ to 29.16 mg L^-1^, and 3.77 mg L^-1^ to 16.09 mg L^-1^ based on WV2, Landsat-8 OLI and MODIS-Aqua sensors respectively. In terms of relative error, MODIS-Aqua, Landsat-8 OLI and WV2 in DA, CA and MTA were 16.96%, 54.09%, and 12.05%, 3.1%, 10.39% and 2.99%, and 2.3%, 4.54% and 4.82% respectively.

S1 – S3 Figs. TSS concentration variability at different spatial resolutions derived from MODIS-Aqua, WV2 and Landsat-8 OLI. (S1) Dredge Area (DA), (S2) Moderate Turbid Area (MTA) and (S3) Clean Area (CA)
